# Editorial: Latest advances in neuroscience at the 9th Croatian Neuroscience Congress

**DOI:** 10.3389/fnmol.2025.1563899

**Published:** 2025-02-06

**Authors:** Kristina Mlinac-Jerkovic, Marija Heffer, Svjetlana Kalanj-Bognar, Senka Blažetić

**Affiliations:** ^1^School of Medicine, Croatian Institute for Brain Research, University of Zagreb, Zagreb, Croatia; ^2^Department of Chemistry and Biochemistry, School of Medicine, University of Zagreb, Zagreb, Croatia; ^3^Department of Medical Biology and Genetics, Faculty of Medicine, Josip Juraj Strossmayer University of Osijek, Osijek, Croatia; ^4^Department of Biology, Josip Juraj Strossmayer University of Osijek, Osijek, Croatia

**Keywords:** neurodevelopment, neurodegeneration, sphingolipids, glioma, CSF, oxytocin, neural stem cells (NSCs), brain injury

Like never before, neuroscience is simultaneously captivating minds as well as decoding them in a thrilling new research era. We are witnesses to sophisticated experimental research tools, augmented by tremendous potentials of artificial intelligence (AI)-assisted technologies and have a pool of knowledge that will hopefully set us on a path to unravel the mysteries of brain function, as well as elucidate complex brain disorders with the aim of improving diagnosis and therapy. With that in mind we held the 9th Croatian Neuroscience Congress. Our aim was to highlight the heterogeneity of our current research, covering diverse fields in neuroscience, so far contributed by some of the pivotal discoveries made by researchers from Croatia or of Croatian origin (Kaur et al., 2025; Kostovic and Rakic, 1990; Rakic, 1971).

This Research Topic includes papers which feature exciting findings and state-of-the art methodologies applied in brain research. Two original research papers address the intricacies of CSF pressure and volume redistribution. Jurjević et al. present data on experimentally caused CSF system impairment in animal model, which indicate that cervical stenosis in a head-up vertical position reduces blood perfusion of the whole brain, while aqueductal obstruction impairs only the perfusion of the local periventricular brain tissue. The reported phenomena give an important **insight into hydrocephalus development**. Strbačko et al. evaluate how body position influences CSF volume redistribution inside the cranial and spinal CSF compartments. The authors performed MRI volumetry in healthy volunteers in three different body positions and report significant CSF volume changes inside the spinal space in the tested body positions, with no significant CSF volume changes inside the cranium in two tested positions. Both papers significantly contribute to the understanding of fundamental physiological processes involved in **CSF dynamics**.

A study examining **regional cerebral oxygen saturation variability in preterm infants** reports a potential predictor or a **marker for increased likelihood of brain injury**. As shown by Caleta et al., preterm infants have an increased aberration of regional cerebral oxygen saturation in early postdelivery period. This was found to be associated with an increased likelihood of brain injury (intraventricular/cerebellar hemorrhage or white matter injury) diagnosis at term-equivalent age. Since timely diagnosis is crucial for treating brain injuries, the results of this study may be used in future management strategies designed to improve neonatal outcomes.

A different study tackles specific aspects of neurodevelopment in young children. Križalkovičová et al. investigate the benefits of judo training in preschool children, 4–7 years old. The authors determined **significant improvements in cognitive and motor performance** in judo-practicing children compared to their non-judo counterparts. Since developmental kinesiology is an emerging field of study, particularly considering the sedentary lifestyle we embraced in the modern age, this finding is especially compelling. Sports encouraging natural movement patterns and proper body control, which is crucial for effective motor performance, will show to be exceptionally important for overall life-long health. Aligned in that direction, a Systematic Review by Szabó et al. sheds light on one particular benefit from sports: **exercise-induced oxytocin**. The authors explored the interplay between oxytocin and exercise. By detailed analysis of a staggering 175 studies, the authors highlight that exercise-induced oxytocin could promote tissue regeneration and has analgesic and anti-inflammatory effects. In addition, it can affect the amount of stress experienced by athletes, and their response to it. Therefore, examining oxytocin's complex interactions with exercise paves the way for future research and application in sports science, psychology, and biomedicine.

This Research Topic is also comprised of articles reporting methodological advances and breakthroughs. A research paper by Prkačin et al. describes a **novel approach to cytoarchitectonics through the use of a supervised neural network prediction algorithm**. The authors defined cortical regions and layers based on clear quantitative criteria and they reveal that the cytoarchitectonic descriptions were reflected in the morphometric measures and cell classifications obtained by the used prediction algorithm. The results of this study suggest that supervised machine learning could aid in defining the morphological characteristics of the cerebral cortex. A study by Lisjak et al. addresses the **treatment for stroke by transplantation of neural stem cells** (NSCs). The authors stereotaxically transplanted NSCs into the stroke affected mouse brains, assessed recovery by MRI and neurological scoring, and evaluated pyroptosis and necroptosis markers. The study shows that NSC transplantation significantly improved neurological recovery compared to control groups, and holds therapeutic potential in stroke recovery by targeting pyroptosis and necroptosis pathways.

**Brain cancer** is also in focus of this Research Topic, specifically diffuse gliomas. A study by Kafka et al., by using immunohistochemistry and methylation specific-PC, reports that **Secreted frizzled-related protein 4 (SFRP4)** expression is reduced in high grade astrocytomas which is not caused by the methylation of its promoter. The study contributes to the recognition of the significance of epigenetic changes in diffuse glioma indicating that restoring SFRP4 protein to its normal expression holds potential as therapeutic avenue. A Mini Review by Karmelić et al. examines the role of sphingolipids in the pathogenesis of highly aggressive adult-type diffuse gliomas. The authors summarize findings on the balance of the pro-apoptotic ceramide and pro-survival sphingosine-1-phosphate (S1P), the so-called **sphingolipid rheostat**. In gliomas, that balance is shifted toward cell survival and proliferation. The authors therefore discuss how targeting the sphingolipid rheostat, through reducing S1P levels or modulating S1P receptors to reduce cell proliferation, as well as through increasing ceramide levels to induce apoptosis, offers a potential therapeutic pathway for glioma treatment. A Perspective paper by Puljko et al. also discusses the link between brain tumors and (glycol)sphingolipids, but from a different aspect. The authors explore the physiological consequences of decreased **GD3 synthase** (GD3S) expression, an enzyme involved in ganglioside biosynthesis. Since GD3S overexpression enhances tumor growth, inhibiting GD3S activity has potential therapeutic effects across different cancer types. However, negative consequences of the inhibition of this enzyme have been underexplored, and the authors highlight them and show original data indicating that inactivated GD3S can generally negatively affect energy metabolism, regulatory pathways, and mitigation of oxidative stress.

A paper by Babić Leko et al. addresses **neurodegeneration**, specifically Alzheimer's disease (AD). The authors analyzed microtubule-associated protein tau (*MAPT*) gene haplotypes and specific *MAPT* haplotype-tagging polymorphisms in more than 900 individuals. Certain polymorphisms were found to be more represented among patients with dementia and apolipoprotein E (APOE) ε4 carriers. Carriers of other specific *MAPT* haplotypes had worse performance on various neuropsychological tests. Hence, these results reveal another **intricate level of interaction between** ***MAPT* haplotypes**, *MAPT* haplotype-tagging polymorphisms, CSF biomarkers, *APOE* genotypes and the development of AD.

An intriguing study from Ratko et al. reports that **guanylate cyclase C (GC-C) expression in human prefrontal cortex depends on sex and feeding status**. The authors determined GC-C protein expression in specific areas of human prefrontal cortex involved in regulation of feeding behavior, as well as in the cerebellar cortex, arcuate nucleus of hypothalamus and substantia nigra in more than 30 brains. This is the first study of GC-C regulation and its possible function in human prefrontal cortex, providing a strong basis for future GC-C studies in human brain, as well as opening new riveting research questions.

Finally, an innovative Hypothesis and Theory article by Stojanovic and Kalanj-Bognar proposes that Toll-like receptors are a **missing link in Notch signaling cascade during neurodevelopment**. Based on data demonstrating Notch and TLR structural engagement and functions during neurodevelopment, along with the authors' description of novel molecular binding models, the authors advocate for the hypothesized role of TLRs in Notch signaling. A truly fresh theory!

In summary, this Research Topic, stemmed from the work presented at the 9th Croatian Neuroscience Congress, is a compendium of various article types, thus providing original findings, fresh perspectives on known topics, systematization of vast data on particular subjects, as well as intriguing and thought-provoking concepts. We hope this will serve as a go-to article collection in the diverse field of neuroscience.
